# Supplementary material for: Geographic weighted regression analysis of hot spots of modern contraceptive utilization and its associated factors in Ethiopia
Source: PLoS One. 2023 Nov 30;18(11):e0288710. doi: 10.1371/journal.pone.0288710 (PMC10688731; doi:10.1371/journal.pone.0288710)
Supplement: Attachment — Submitted filename: Response to Reviewers.rtf [file pone.0288710.s002.rtf]

Response to Reviewers
Reviewers' comments: 
The reviewer comments that your methodology section requires further clarification and that your tables should report confidence intervals in the percentage column. In addition, the reviewer comments that the discussion second of your study requires more information, and the conclusions need to be reported better. 
 Author’s Responses to comments
Extensive revisions were made in methodology, discussion and conclusion sections. 
Table 1&2 indicates socio-demographic characteristics or descriptive analysis of the study participants. So, no need of writing confidence interval in the percentage column. But confidence intervals were reported in the statistical analysis or in the multilevel analysis table.
Reviewers' comments: 
In addition to the reviewer's comments, it has come to the editorial office's attention that there appear to be a lot of grammar, spelling, and typological errors in your manuscript. For example, the abstract reads "Therefore, the this study aimed to investigate" as well as "public health interventions targeting colds pot areas of modern contraceptive considering rural residents". Please make sure to carefully review the writing of the manuscript. 
 Author’s Responses to comments
Grammar, spelling, and typological error were observed significantly in our manuscript and we tried to correct as far as our knowledge. 
Reviewers' comments: 
Furthermore, it has come to the editorial office's attention that in Editorial Manager, the submission details of your manuscript only list one author, whereas your title page suggests 6 authors contributed to this article. Please make sure that you register each author on your manuscript correctly. Please note that the journal will follow up with each author individually to confirm that they know of, and consent to the submission of this manuscript. 
Author’s Responses to comments 
Only corresponding authors were registered mistakenly in our previous submission. But all authors were registered in our revised manuscript submission. 
Reviewers' comments: 
5. We note that Figure 4, 6, 7, 8, 9, 10 and 11 in your submission contain [map/satellite] images which may be copyrighted. All PLOS content is published under the Creative Commons Attribution License (CC BY 4.0), which means that the manuscript, images, and Supporting Information files will be freely available online, and any third party is permitted to access, download, copy, distribute, and use these materials in any way, even commercially, with proper attribution. For these reasons, we cannot publish previously copyrighted maps or satellite images created using proprietary data, such as Google software (Google Maps, Street View, and Earth). For more information, see our copyright guidelines: http://journals.plos.org/plosone/s/licenses-and-copyright.
 
We require you to either (1) present written permission from the copyright holder to publish these figures specifically under the CC BY 4.0 license, or (2) remove the figures from your submission:
Author’s Responses to comments
Dear reviewers, we would like to assure you that all figures in our manuscript is our original work which are produced using our data set in ArcGIS version 10.7 and SaTScan version 9.6 software and it is a part of spatial epidemiology course which is our specialization area as an epidemiologist. So, figures are not copyrighted or satellite image.
As we stated in the methodology part “The survey data was accessed from the measure Demographic and Health Survey (https://dhsprogram.com/).”  Only EDHS 2016 data set with it is shape file was requested and received online using (https://dhsprogram.com/) this address.
Reviewers' comments: 
6. Please upload a copy of Figure S1, to which you refer in your text. If the figure is no longer to be included as part of the submission please remove all reference to it within the text.
Author’s Responses to comments
All reference were removed from our text because the figure is no longer to be included as part of our manuscript submission.
Reviewers' comments: 
7. Thank you for submitting the above manuscript to PLOS ONE. During our internal evaluation of the manuscript, we found significant text overlap between your submission and the following previously published works, some of which you are an author.
Author’s Responses to comments
Significant text overlapping was observed during internal evaluation of our manuscript and revisions were made in our manuscript by rephrasing the duplicated text. Furthermore, ideas were incorporated that explain how the current manuscript advances on previous studies.
 Reviewers' comments: 
Reviewer #1: Reviewer report
Introduction
Line 118-123… it could be good if this paragraph shows what are the gaps in the literature and what would this research is intended to address.
Author’s Responses to comments
This paragraph (line 118-123) already replaced by paragraph that shows the gaps in the previous literature and the gaps intended to be addressed by this study. 
 Reviewers' comments: 
Methods
Line 151.. ‘…The study population was all Women who had any modern contraceptive in the five years…’ it is not clear; are you considered only those who had used contraceptive in the last five years?
The inclusion and exclusion criteria were not clear. Did you include or exclude sexually active women?
Author’s Responses to comments
Study population, inclusion and exclusion criteria were cleared with revision based on our study participant selection.
 Reviewers' questions: 
Line 197… what is the national median value?
Author’s Responses to comments
Ethiopian Demographic Health Survey (EDHS) were considered as representative data at national level. Since, we don’t have any standard to classify the community wealth status as high and low community poverty; we generated the national median value (.3333333) using the household wealth index in each enumeration areas. After checking the normality of the distribution the median value was taken to classify high/low poverty status of the community, since the distribution is not normal.  
 Reviewers' questions: 
Line 200… what are your bases to define Media exposure in such a way? it is the standard definition? If so, cite a reference
Author’s Responses to comments
Operational definition of media exposure was taken from Ethiopian Demographic Health Survey (EDHS) manual, which is considered as standard definition and the reference cited.
 Reviewers' questions: 
Line 240… one of the issues with GWR is multicollinearity, did the authors check multicollinearity?
Author’s Responses to comments
Multicollinearity was checked and already reported under table 3 during Geographic Weighted Regression (GWR) analysis using variance Inflation Factor (VIF) as a default result reported during GWR analysis in ArcGIS software. 
 Reviewers' questions: 
Results
Table 1, which religions are grouped under the ‘other’ category? Add confidence intervals for the percentage column.
Author’s Responses to comments
Religions under other religion categories are: Catholic and traditional
Table 1&2 indicates socio-demographic characteristics or descriptive analysis of the study participants. So, no need of writing confidence interval in the percentage column. But confidence intervals were reported in the statistical analysis or in the multilevel analysis table.
 Reviewers' questions: 
What is the relevance of figure 3 as the values already available in table 2?
Author’s Responses to comments
Comment accepted and figure 3 was removed since it is redundant 
 Reviewers' questions: 
Figure 6 reported three confidence levels (90%, 95%, and 99%), I suggest using only one confidence level.
Author’s Responses to comments
Three confidence levels are a default in ArcGIS software to report hot spot analysis. But the readers can use their preference level of confidence interval. 
 Reviewers' questions: 
Line 400-415. I would suggest restating this paragraph as the authors used the one phrase ‘… there is negative relationship between proportion of…’ again and again.
Author’s Responses to comments
Comment accepted and the paragraph restated 
 Reviewers' questions: 
Table 5… how did you define /measure the distance from the health facility? What does big problem and not problem mean?
Author’s Responses to comments
Distance from home to health facility took >30min on foot considered as big problem and distance from home to health facility took <30min on foot considered as not big problem
 Reviewers' questions: 
Table 5… In EDHS there are five categories for wealth index. Why did you have 3 categories of wealth index?
Author’s Responses to comments
Most of the time many scholars use three categories of wealth index, in Ethiopian context five categories of wealth index doesn’t give sense. For example, the gap between poorest and poorer, richer and richest is not well understandable in our community. So, authors prefer to use three categories of wealth index not to confuse our readers.  
 Reviewers' questions: 
Table 5. Why Tigray region selected as a reference?
Author’s Responses to comments
Tigray region selected as reference because of better health infrastructure and better health service coverage in the country. 
 Reviewers' questions: 
Discussion
Line 525… “The highest modern contraceptive utilization was recorded in the Amhara region…”. This statement contradicts your result- contraceptive prevalence was higher in Oromia region (37%) than Amhara region (25%)
Author’s Responses to comments
This was typing error and already corrected  
 Reviewers' questions: 
Line 507-511… “…. Other religion followers were found to be less likely to use modern contraceptive methods in Ethiopia. This result is consistent with other study done in Zambia[43]. The possible reason might be due to the fact that religion might have similar socio-cultural importance in influencing the life of women in Ethiopia. Especially, the introduction of some family planning teachings in religiously conservative societies might be disadvantageous……”.
This paragraph is not clear. Which religions were grouped under ‘other region followers’? Be cautious to compare these variables in Ethiopia with Zambia.
Author’s Responses to comments
List of religions under “other” categories are Catholic and traditional as it’s explained previously. When we compare these variables between Ethiopia and Zambia, we understand that from different literatures there is similarity between two countries community in the aspect of religion. 
 Reviewers' questions: 
Conclusion
What is your basis to conclude the prevalence of modern contraceptives in Ethiopia was low?
It is not informative stating as a hotspot and cold spot, as these are not defined before.
Author’s Responses to comments
The current prevalence of modern contraception in Ethiopia was low as we compared with the national target which is should be >80%.
 
