# Supplementary material for: Geographic weighted regression analysis of hot spots of modern contraceptive utilization and its associated factors in Ethiopia
Source: PLoS One. 2023 Nov 30;18(11):e0288710. doi: 10.1371/journal.pone.0288710 (PMC10688731; doi:10.1371/journal.pone.0288710)
Supplement: Attachment — Submitted filename: review.docx [file pone.0288710.s003.docx]

### Dear editors for PLOS ONE

Thank you for allowing me to review this paper. The paper is addressed important public health topic. I have provided some question and comments as outlined below.

**General question**

1. What is the need of doing another research since there is a similar study done in Ethiopia?
2. What will it add to the already known facts?
3. What was the gap in the previous study?
4. Why you select multilevel robust Poisson analysis for this study?

**General comment**

I. Title: In the title you should remove the methods that you used
II. **Introduction section**: I would like to thank you, your introduction is very attractive and well written. While, it is better to include consequences of lack of modern contraceptive utilization.
III. **Method section**: it is too lengthy & try to summarized sentence especially, your method of analysis like spatial analysis part. What are the assumption that you have checked In OLS model? Please write shortly and state in method section.
IV. **Result section**: Dhs measure education as o education, primary, secondary, and higher. Why did you merge secondary ad higher? You could have merged it only if the distribution of one was extremely small, which is not the case. In addition, did you focus only on married women? If you did not, what about women who were single?

**From li**ne number 306-312 already reported in the hotspot analysis section, so please try to remove this paragraph

A beauty of this study is the large data and the multilevel analysis performed. However, you did not include in the background, the importance of the community factors that warranted a multilevel analysis. Overall, the discussion is shallow and is a repetition of the results. Furthermore, it needs some work on language.
