# Supplementary material for: Geographic weighted regression analysis of hot spots of modern contraceptive utilization and its associated factors in Ethiopia
Source: PLoS One. 2023 Nov 30;18(11):e0288710. doi: 10.1371/journal.pone.0288710 (PMC10688731; doi:10.1371/journal.pone.0288710)
Supplement: Attachment — Submitted filename: Response to Reviewers.docx [file pone.0288710.s004.docx]

**Response to Reviewers**

**Reviewers' comments:**

**Journal Requirements:**

Please review your reference list to ensure that it is complete and correct. If you have cited papers that have been retracted, please include the rationale for doing so in the manuscript text, or remove these references and replace them with relevant current references. Any changes to the reference list should be mentioned in the rebuttal letter that accompanies your revised manuscript. If you need to cite a retracted article, indicate the article’s retracted status in the References list and also include a citation and full reference for the retraction notice**.**

**Author’s Response**

**Dear Sir/Madam,** I would like to assure you that my reference is complete and correct also no cited paper that has been retracted.

**Reviewer question**

1. What is the need of doing another research since there is a similar study done in Ethiopia?

**Author’s Response**

The previous study was conducted only considering married women as a study population and without considering of geographical variation of variables across Ethiopian regions. But there is evidence that indicates variation of variables influence among regions in health service utilization and unmarried women are sexually active almost the same as married women[20]. Therefore, this study aimed to investigate spatial regression analysis of modern contraceptives use among women of reproductive age in Ethiopia, irrespective of their marital status and identify the potential factors associated with the use of modern contraceptives and considering geographic variation of variables. As a result this study result will facilitate evidence based decision making by complementing limitations of the previous studies.

**Reviewer question**

2. What will it add to the already known facts?

**Author’s Response**

Findings of this study will be useful for health planners, policymakers, and non-governmental partners who are working to improve the health and well-being of women of reproductive age in Ethiopia. Besides, mapping hotspot areas of modern contraceptive use, it will provide a deeper understanding of the impacts of already implemented interventions in each region of the country. Furthermore, it will assist in designing programs and strategies to increase coverage, quality, and equity of modern contraceptive service utilization and it is determinant factors at regional level.

**Reviewer question**

3. What was the gap in the previous study?

**Author’s Response**

The previous study was conducted only considering married women as a study population and without considering of variation of variables across Ethiopian regions (Geographical weighted regression analysis not done).

**Reviewer question**

4. Why you select multilevel robust Poisson analysis for this study?

**Author’s Response**

In our study the ICC shows that there is a significant clustering effect (ICC>10%). In this case, the prevalence ratio is the best measures of association because reporting the odds ratio will result over estimation of association between modern contraception use and it is predictors.

**Reviewer general comment**

I. Title: In the title you should remove the methods that you used

**Author’s Response**

Dear reviewer, I thank you for your constructive comment and already I have removed it

II. Introduction section: I would like to thank you; your introduction is very attractive and well written. While, it is better to include consequences of lack of modern contraceptive utilization.

**Author’s Response**

Dear reviewer, first of all, thank you for your detail review and comment, but consequences of lack of modern contraceptive utilization was mentioned between line number 87 and 91 as a positive statement or “the advantage of the modern contraceptive use among reproductive age is very vital for designing interventions, plans, and policies to address premature age pregnancies and other related issues. It is also useful to reduce unsafe abortions, maternal death, and sexually transmitted infections (STI)”. I left listing consequences of lack of modern contraceptive utilization intentionally in order to avoid idea redundancy.

**Reviewer general comment**

III. Method section: it is too lengthy & try to summarized sentence especially, your method of analysis like spatial analysis part. What are the assumptions that you have checked In OLS model? Please write shortly and state in method section.

**Author’s Response**

Accepted and corrected

**Reviewer general comment**

IV. Result section: Dhs measure education as o education, primary, secondary, and higher. Why did you merge secondary ad higher? You could have merged it only if the distribution of one was extremely small, which is not the case. In addition, did you focus only on married women? If you did not, what about women who were single? From line number 306-312 already reported in the hotspot analysis section, so please try to remove this paragraph A beauty of this study is the large data and the multilevel analysis performed. However, you did not include in the background, the importance of the community factors that warranted a multilevel analysis. Overall, the discussion is shallow and is a repetition of the results. Furthermore, it needs some work on language.

**Author’s Response**

Number of participants with higher educational status is extremely low and failed to satisfy the Chi-square assumption that why we decide to merge them with secondary education. As we have clearly stated under the inclusion criteria all Women in the reproductive age group were included in the study irrespective of their marital status and this is one of the uniqueness of our study from previous studies.

Dear reviewer, result reported from line number 306-312 and ideas reported under in the hotspot analysis section are different. OLS regression results were reported from line number 306-312 and may be regression results consistent with hotspot analysis since it is statistical evidence. So, it is statistical evidence that strengthening our hot spot analysis and indicating our result is consistent.

Sentence which states about the importance of the community level factors that warranted a multilevel analysis included under introduction section
